# Bioactive Fish Scale Scaffolds with MSCs‐Loading for Skin Flap Regeneration

**DOI:** 10.1002/advs.202201226

**Published:** 2022-05-22

**Authors:** Xiang Lin, Bin Kong, Yujuan Zhu, Yuanjin Zhao

**Affiliations:** ^1^ Department of Rheumatology and Immunology Nanjing Drum Tower Hospital School of Biological Science and Medical Engineering Southeast University Nanjing 210096 P. R. China; ^2^ Oujiang Laboratory (Zhejiang Lab for Regenerative Medicine Vision and Brain Health) Wenzhou Institute University of Chinese Academy of Sciences Wenzhou 325001 P. R. China; ^3^ Institute for Stem Cell and Regeneration Chinese Academy of Science Beijing 100101 P. R. China

**Keywords:** biomaterial, fish scale, MSC, skin flap, tissue regeneration

## Abstract

Skin flap transplantations are common methods for covering and repairing tissue defects in surgery, while the survival rates of these skin flaps are still low due to the vascular crisis and necrosis. To improve this situation, herein a novel biohybrid scaffold is proposed by integrating the advantages of anisotropic fish scales and mesenchymal stem cells (MSCs) for skin flap regeneration. The fish scale scaffold is obtained through its decellularization and decalcification processes, which reserved intact collagen, glycosaminoglycan, and other endogenous growth factors for MSCs and human vascular endothelial cells proliferation. As the scaffold maintains intrinsic anisotropic structures on both surfaces, the proliferative cells can be elongated along the aligned structures on the fish scale, which endow them the capacity to differentiate into multiple directions. Based on these features, it is demonstrated from an in vivo experiment that the MSCs‐loading fish scale scaffolds can effectively convert the activated inflammatory macrophages into anti‐inflammatory properties, reduce the inflammation around the flap, and improve the survival rate. These results indicate that the MSCs‐loading fish scale scaffold is suitable and has the potential for skin flap regeneration and functional recovery.

## Graphical abstract

Bioactive fish scale scaffolds present highly oriented collagen structures，and a varitey of cells can proliferate well on the surface of the scaffold. MSCs (mesenchymal stem cells)‐loading fish scale scaffolds could effectively promote angiogenesis to improve the survival rate in random flap. These results indicate that the MSCs‐loading fish scale scaffolds has potential for tissue regeneration.

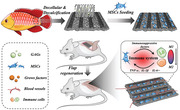

## 1 Introduction

Random skin flaps are often used in the reconstruction of tissue defects caused by trauma, tumor, infection, and other metabolic diseases.^[^ ^1^ ^]^ However, serious complications such as vascular crisis and necrosis may occur after surgery, which could cause the formation of chronic wounds and repeated operations.^[^ ^2^ ^]^ Therefore, it is important to promote the vascularization of the flap to enhance its survival rate. In this aspect, many studies have demonstrated that skin flap could be repaired by applying tissue engineering methods to implant 3D scaffolds with functional cells and growth factors into the distal part of the flap.^[^ ^3^ ^]^ Although such attempts have fabricated scaffolds with controllable biodegradation rate and mechanical strength, the immune rejection after implantation in vivo is still fundamentally challenging.^[^ ^4^ ^]^ In addition, the performance and bioactivity of these scaffolds generated from natural or synthetic materials is still limited for simulating the complete extracellular matrix (ECM) environment and complex physical structure of tissues.^[^ ^5^ ^]^ Moreover, the existing scaffolds rarely have an anisotropic micro‐nano structure that can induce the directional growth of cells, which is not conducive to enable the specific function of tissues with anisotropic ECM, such as muscle, blood vessel, nerve, etc.^[^ ^6^ ^]^ Thus, seeking for new bioactive scaffolds as tissue engineering materials for skin flap regeneration is still a clinical challenge.

**Figure 1:**
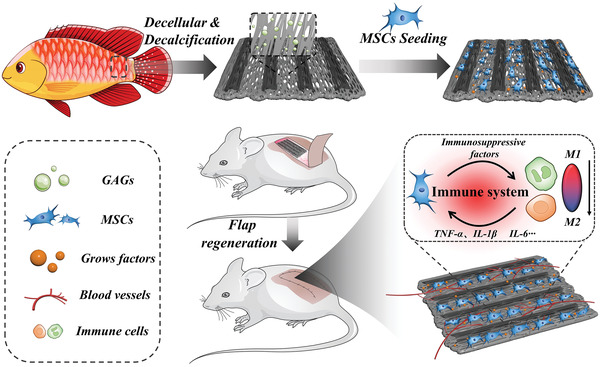
Scheme of the fabrication and skin flap survival application of the MSCs‐loading fish scale.

In this paper, we reported a novel biohybrid scaffold by integrating the advantages of anisotropic fish scales and MSCs for skin flap regeneration, as schemed in **Figure** **1**. As efficient natural dermal armors, fish scales are usually composed of minerals (hydroxyapatite as main component) and multiple layers of collagen fibers to form a laminated plywood structure.^[^ ^7^ ^]^ Such composition and structure endow the fish scale with excellent biocompatibility and sufficient mechanical strength.^[^ ^8^ ^]^ Besides, as a kind of pluripotent stem cell with self‐renewal and multidirectional differentiation ability, the transplanted mesenchymal stem cells (MSCs) can differentiate into fibroblasts, myofibroblasts, vascular endothelial cells, and other resident cells of regional tissues to promote tissue repair.^[^ ^9^ ^]^ However, the current stem cell transplantation methods have obvious shortcomings. For example, most of the injected stem cells are difficult to reach the diseased target tissues or organs.^[^ ^10^ ^]^ In addition, these stem cells with limited numbers are difficult to retain in the injured site for a long time, which make it difficult to guarantee the effectiveness of treatment. Actually, compared with injected method, scaffolds with aligned structures can provide distinctive morphology to guide the proliferation and differentiation of MSCs.^[^ ^11^ ^]^ Therefore, we believe that the employment of fish scale scaffold to deliver MSCs to the injured site provides a new approach for the regeneration of skin flap.

To implement this concept, we seeded MSCs on decellularized and decalcified fish scales with oriented structures to construct 3D cell‐loading scaffolds for promoting the skin flap regeneration. By employing sodium dodecyl sulfate (SDS) and nitric acid, the cellular contents and the mineralized layer of fish scale scaffold were almost completely cleared. Besides, as the decalcified fish scale scaffold reserved intact collagen, glycosaminoglycan, and other endogenous growth factors, both MSCs and human vascular endothelial cells (HUVECs) could proliferate well on the surface of the fish scale scaffold. In addition, because the resultant fish scale scaffold maintained highly oriented collagen structures, which was very similar to the dermal tissue, they could induce the alignment growth of MSCs and HUVECs compared with these cells seeded on the glass plate. Based on these features, we have demonstrated in an in vivo experiment that the MSCs‐loading fish scale scaffolds could effectively promote the angiogenesis, regulate the polarization of macrophages to the M2 phenotype, reduce the inflammation around the flap and the necrosis area, and thus improve the survival rate in random flap. All of these results indicated that the MSCs‐loading fish scale scaffolds provides a new prospect for tissue regeneration.

## 2 Results and Discussion

**Figure 2:**
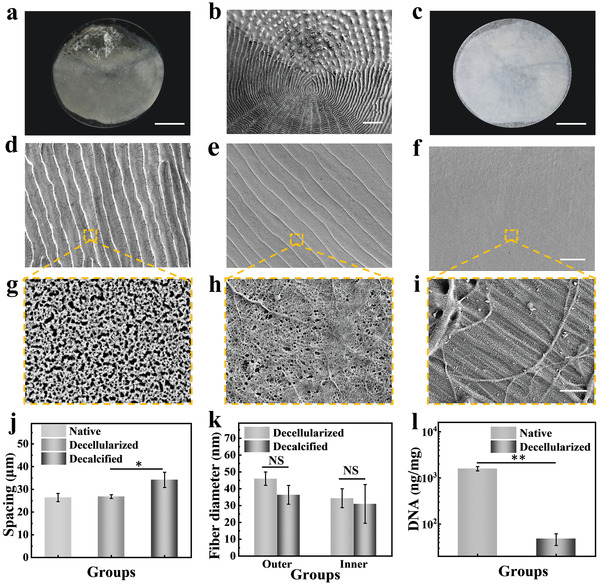
a) Optical image of the native fish scale. b) SEM image of the outer surface of fish scale. c) Optical image of the decellularized fish scale. d) SEM images of the outer surface in the decellularized fish scale and g) the magnified field. e) SEM images of the outer surface in the decalcified fish scale and h) the magnified field. f) SEM images of the inner surface in the decalcified fish scale and i) the magnified field. j) The spacing of micro channel on the surface of the fish scale after different treatments (*n* = 5). k) The fiber diameter on the surface of the fish scale after different treatments (*n* = 5). l) DNA content in native and decellularized fish scale (*n* = 5). The scale bars are 5 mm in b), 200 µm in c), 200 nm in i). The error bar represents standard deviation, NS: no significant, * *p* < 0.05, ** *p* < 0.01.

In a typical experiment, fish scales were decellularized and decalcified by SDS and nitric acid prior to cell culture. All of native fish scales had similar basic surface structures, which consisted of embedded parts and overlapping parts. Due to the covering of fish skin, the fish scale presents an optically opaque nature. About one‐third of the scales were embedded in the dermis, and about two‐thirds of the back surface overlapped with adjacent scales (**Figure** **2**a; and Figure S1, Supporting Information). In the overlapping area, a series of microchannel‐like structures were arranged in a regularly ordered and repeating pattern (Figure 2b). Besides, the outer surface of fish scale had a thin mineralized layer, which formed a randomly oriented network. From the cross‐sectional scanning electron microscope (SEM) image of the scales we found the collagen fibers were arranged parallelly in each layer, and there was also a certain angular offset between the different layers (Figure S2, Supporting Information). After decellularization process, the dermis layer on the surface of the embedded parts was removed, and the entire fish scale turned into transparent (Figure 2c), but the calcified layer was still retained (Figure 2d,g). In order to remove this mineralized layer, nitric acid was used in the decalcification process and the decalcification time was further optimized to 8 h (Figure S3, Supporting Information). After decalcification, the outer surface of the fish scales still retained the oriented microstructure (Figure 2e), and the magnified image showed nanoscale collagen fibers exposed on the decalcified surface (Figure 2h). Besides, the channel spacing became larger than native and decellularized fish scale due to the removal of mineralized layer (Figure 2j). Furthermore, the inner surface also showed a smooth and oriented collagen structure after decalcification process (Figure 2f,i), and the fiber diameter did not change significantly (Figure 2k). These results indicated that the process of surface treatment did not damage the original collagen structure of fish scales. Besides, there was no residual cell nucleus on the surface of the fish scales after decellularization (Figure S4, Supporting Information), and the residual amount of DNA was lower than the minimum standard for decellularized scaffolds (50 ng mg^−1^) (Figure 2l).

**Figure 3:**
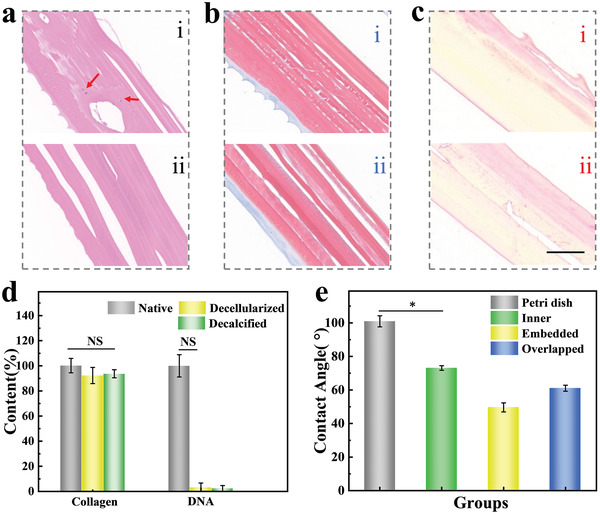
a) H&E staining of the fish scale before (i) and after (ii) decalcification, the red arrows indicated the residual nuclei. b) Masson staining of the fish scale before (i) and after (ii) decalcification. c) Sirius Red staining of the fish scale before (i) and after (ii) decalcification. The scale bar is 200 µm. d) Quantification of collagen and DNA content in native fish scale, decellularized fish scale and decalcified fish scale. e) Water contact angle in petri dish and different sections of fish scale (*n* = 5 for each group). The error bar represents standard deviation, NS: no significant, * *p* < 0.05.

Fish scales are composed of layers of oriented collagen fibers, which are ideal candidates for 3D tissue engineering materials. As a kind of ECM, fish scales also provide the necessary conditions for cell growth, including the structure provided by the complex mesh of collagen and elastin fibers, various growth factors, and so on. Hematoxylin and eosin (H&E) images showed that most cells in the cross section were also removed after decellularization (**Figure** **3**a,d). The Sirius Red and Masson staining results of the cross‐section of fish scale indicated that collagen fibers mainly distributed on the surface layer and arranged in a unique structure. In addition, the collagen fibers had no obvious structural changes before and after decalcification (Figure 3b–d). In addition to collagen, fish scale is known as a rich source of glycosaminoglycans (GAGs), which are of critical importance in intercellular communication and physiological processes. Then, in order to determine the changes in GAGs content, the fish scale samples were stained with Alcian blue, and the results demonstrated that GAGs content did not decrease significantly due to the short pretreatment time (Figure S5, Supporting Information). These findings suggested that the processed fish scale could retain the bioactive GAGs, and potentially provide an ideal environment for cell culture.

Proper hydrophilicity could enhance the initial attachment of cells to the scaffold and subsequently improve cell growth and function. As expected, the fish scales were more hydrophilic than the petri dish, and no significant changes were observed in the hydrophilicity of the fish scales before and after treatment (Figure 3e; and Figure S6, Supporting Information). Therefore, we can expect that the structure and chemical properties of the decalcified fish scales are conducive to the growth of cells, making them potentially useful in the field of biomedicine. In order to further evaluate the influence of the decellularization process of the fish scale scaffold in terms of mechanical properties, the material was cut into rod tensile samples, and tensile experiments were performed in axial and transverse directions, respectively. The resulting stress–strain curve (Figure S7a,b, Supporting Information) showed an initial quasilinear region. The material softened slightly before the stress reached to 40–50 MPa (depending on the proportional direction), after which the stress dropped significantly. Furthermore, difference between native fish scale and the decalcified specimens was small during the stretching process in the same direction. This was because the calcified layer was only a limit part of the fish scale scaffold and the collagen fibers were not destroyed in the pretreatment process. Therefore, the mechanical properties did not decrease significantly after decalcification process. In addition, the ultimate strength and stress in different directions were shown in Figure S7c,d (Supporting Information). The strength in the axial direction was generally higher than that in the transverse direction, which indicated that the tensile property of the fish scale scaffold showed anisotropic feature. In addition, samples in different directions had similar elongation at break which indicated that there was no significant difference in total elongation in different directions.

**Figure 4:**
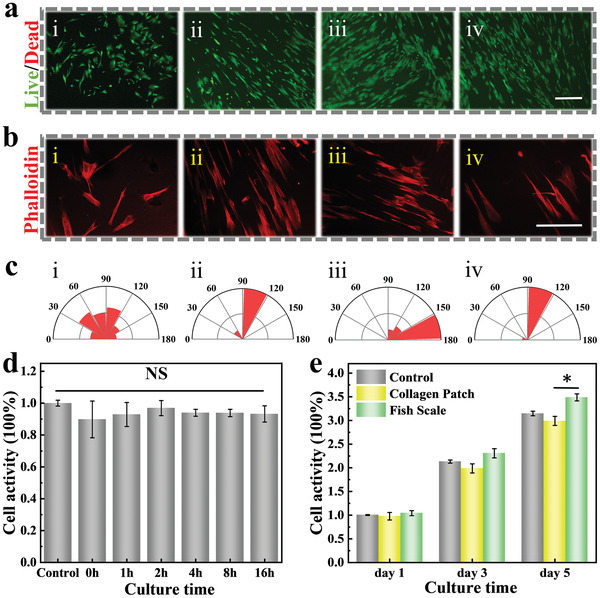
a) Fluorescence images of MSCs planted on (i) petri dish, (ii) inner surface, (iii) and (iv) overlapped area of the fish scale, the scale bar is 100 µm. b) Representative phalloidin staining images on (i) petri dish, (ii) inner surface, (iii) and (iv) overlapped area of the fish scale, the scale bar is 50 µm. c) Quantitative statistics of the cells angle in each group. d) Relative activity of 3T3 cells after different decalcification time (*n* = 5). e) Relative cell proliferation activity of MSCs on different materials (*n* = 5). The error bar represents standard deviation, NS: no significant, * *p* < 0.05.

Generally, high biocompatibility is required for excellent scaffolds in tissue engineering. Since nitric acid was used for decalcification of the fish scale scaffold in the pretreatment process, the biocompatibility of fish scale scaffold is then verified. For this purpose, we conducted 3‐(4,5‐dimethylthiazol‐2‐yl)‐2,5‐diphenyltetrazolium bromide （MTT） experiments to study the metabolic activity of 3T3 fibroblasts after gradient processing for different time periods. The results showed that the nitric acid treatment process did not affect the biological activity of the scaffold (**Figure** **4**d). In vivo, vascular endothelial cells play an important role in angiogenesis and revascularization. Thus, HUVECs were cultured on the fish scale scaffold and stained by Calcein/AM after 3 days. The results showed that cells grew well on the scaffold with oriented morphology (Figure S8a, Supporting Information), suggesting the great potential of the fish scale as the scaffold for oriented cell culture. Currently, many approaches to fabricate anisotropic materials need complex processing methods such as aligned electrospinning, directional freezing, micropatterns, which will lead to disputes about the costs and biocompatibility of these materials. Therefore, cell conditions were further examined by MTT experiment. The quantitative analysis has displayed the enhanced cell adhesion and proliferation with fish scale in comparison to petri dish (Figure S8b, Supporting Information).

As present, increasing studies have demonstrated the great potential of MSC in regenerative medicine. Hence, MSCs were utilized in this study and first seeded on the surface of generated scaffolds. The cells seeded on the petri dish showed irregular shape arrangement. On the contrary, MSCs spread and closely contacted with each other on the scaffold after adherence (Figure 4a). In addition, phalloidin staining proved that the cells grew on the fish scale scaffold had a more elongated structure (Figure 4b), and by counting the cytoskeletal orientation we found that cells could grow directionally along the microchannel (Figure 4c), which indicated that the oriented structures can effectively induce cell elongation and cell infiltration. We also found that the MSCs proliferated better on the fish scale scaffold in comparison with the regular collagen patch (Figure 4e). Therefore, these results indicated that the MSCs‐loading fish scale scaffold had the potential for the in vivo experiments. In addition to biocompatibility, degradability is also an important parameter for biomaterials used in in vivo experiment. Next, the degradation of fish scale scaffold in 28 days was studied. As shown in Figure S9 (Supporting Information), the fish scale scaffolds were difficult to be observed after transplantation for 28 days. However, the disk scaffolds made of 10% poly(ethylene glycol) double acrylate（PEGDA） in the control group still retained the initial shape, which indicated that the fish scale scaffolds as biological materials had good degradation properties for in vivo experiments.

**Figure 5:**
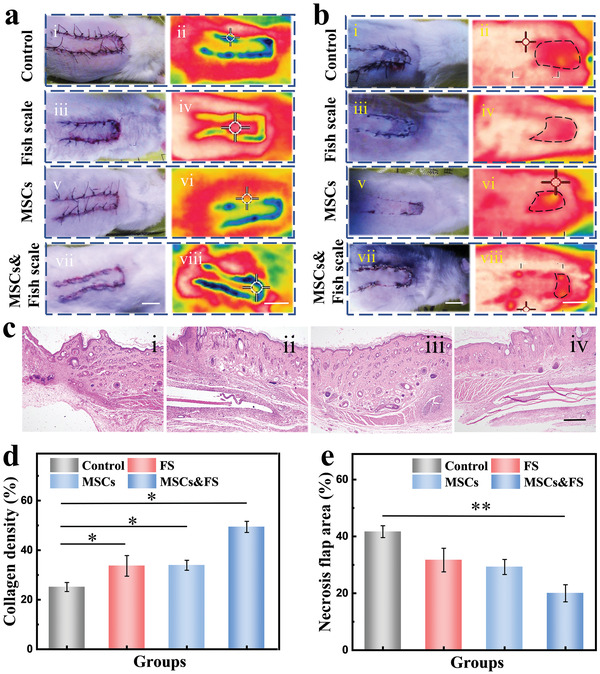
a) The optical images of flap necrosis area in different groups (i, iii, v, vii) 8 h after operation and their corresponding infrared thermal images (ii, iv, vi, viii), the scale bar is 1 cm in (vii) and (viii). b) The optical images of the flap necrotic area in different groups (i, iii, v, vii) on day 7 and their corresponding infrared thermal images (ii, iv, vi, viii), the scale bar is 1 cm in (vii) and (viii). c) The H&E stained images of skin tissue near the area of skin flap necrosis in (i) control group, (ii) FS group, (iii) MSCs group, (iv) MSCs&FS scaffold group, the scale bar is 500 µm. d) Statistics of collagen deposition density in different groups (*n* = 4). e) Statistics of the area of skin flap necrosis in different groups (*n* = 4). The error bar represents standard deviation, * *p* < 0.05, ** *p* < 0.01.

The skin flap is often necrotic due to insufficient vascular circulation in skin flap transplantation. MSCs and ECMs have important biological properties of tissue repair and regeneration, and also have the great ability to promote vascular regeneration. Thus, we studied the potential of Fish scale (FS), MSCs, and MSCs‐loading fish scale (MSCs&FS) scaffolds to treat random flap necrosis. For this purpose, a flap model of ≈5 cm long was created on the back of the Sprague‐Dawley (SD) rat and used to evaluate the survival conditions of the flaps in different groups. The flap wounds in all groups were well closed without obvious necrosis in 8 h after operation (**Figure** **5**a). However, various degrees of distal flap necrosis occurred in each group on day 7 after surgery. The dark area of the skin flap represented the necrotic area, which were further verified by the thermal imaging (Figure 5b). Besides, the area of the necrotic skin flap was significantly reduced in experimental groups, and the MSCs&FS group revealed the lowest necrosis flap area (Figure 5e). Although H&E staining showed a small amount of scaffold remained in the MSCs&FS scaffold group, there was no obvious necrosis of the skin around the scaffold (Figure 5c). In addition, collagen deposition is a key parameter for tissue regeneration, and thereby collagen density was assessed in different groups. Since both MSCs and ECM could promote tissue regeneration, more collagen deposition was shown in the experimental group (Figure 5d). Notably, the degree of collagen alignment around the scaffold group was also improved in the FS group and MSCs&FS group, due to the anisotropic structure of the scaffold (Figure S10, Supporting Information). These results have suggested the great potential of MSC and fish scale scaffold to promote the reconstruction of tissue structure.

**Figure 6:**
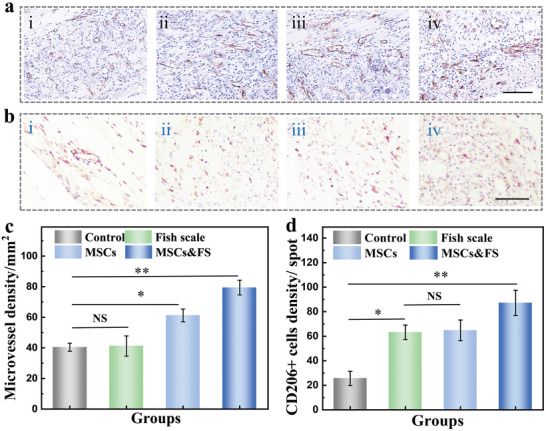
a) Representative CD31 immunohistochemical staining pictures of i) control groups, ii) FS group, iii) MSCs group, iv) MSCs&FS group, the scale bar is 100 µm. b) Representative CD206 immunohistochemical staining pictures of i) control groups, ii) FS group, iii) MSCs group, iv) MSCs&FS group, the scale bar is 50 µm. c) Quantitatively study of the number of microvessels surround the flap in different groups (*n* = 4). d) Number of positive M2 type macrophages in each group (*n* = 4). The error bar represents standard deviation, NS: no significant, * *p* < 0.05, ** *p* < 0.01.

In order to further explore the reasons why MSCs and fish scale scaffolds had a therapeutic effect on promoting the survival of skin flaps, we focused on the angiogenesis of the skin flap. Angiogenesis is of great significance to the survival of skin flaps, therefore, immunohistochemical staining were then conducted. As shown in **Figure** **6**a, immunohistochemical staining of vascular marker CD31 showed the angiogenesis between the skin flap and the wound bed in different experimental groups. Compared to the control group, the blood vessel content surrounding the dermal tissue was significantly increased in experimental groups (Figure 6c), which indicated that both FS scaffold and MSCs could promote angiogenesis. The survival of MSCs after implantation was important, and green fluorescent protein (GFP)‐MSCs were employed to study the survival of MSCs in vivo. The results demonstrated that MSCs seeded on FS scaffolds showed longer survival time, which enhanced the ability of MSCs to promote vascularization and remodel the immune microenvironment in vivo (Figure S11, Supporting Information). Although MSCs&FS scaffold showed ideal ability in promoting vascularization, further evaluation of the inflammatory response induced by implanted scaffold and MSCs was also important. The inflammatory response in the experiments was investigated by immunohistochemical staining for macrophage and monocyte marker CD68 (Figure S12a,c, Supporting Information). The implantation site in FS group showed limited macrophage aggregation response with the implantation of fish scale scaffold, but there was no significant difference compared to the control group. Besides, the content of macrophages in both MSCs and MSCs&FS groups was significantly decreased due to the presence of MSCs. These results suggest that MSCs can effectively reduce the inflammatory response around the necrotic area of the skin flap.

In inflammatory response, M2 macrophage has a positive effect on tissue regeneration. The research has found that both ECM and MSCs could facilitate the polarization of macrophages toward the M2 phenotype. To further identify the types of macrophages, immunohistochemical staining for M2 macrophage marker CD206 was conducted to access the number of M2 phenotype macrophages (Figure 6b,d). We found that all of the experimental groups showed more M2 phenotype macrophages than the control group in wound sites, and the MSCs&FS scaffold group showed the largest population in M2 phenotype macrophagesand. Besides, the number of M2 phenotype macrophages between the fish scale group and MSCs group had no significant difference. This suggested a positive effect of both MSCs and fish scale on the polarization of macrophages to the M2 phenotype during tissue regeneration. Furthermore, the M1 phenotype marked by iNOS‐positive macrophages showed an opposite trend. These results indicated that the MSCs‐loading fish scale scaffolds had the great potential to promote the survival of random skin flap.

## 3 Conclusion

In this paper, we proposed a novel approach based on fish scale with anisotropic structure to promote the random skin flap regeneration. Through the decellularization and decalcification process, the residual GAGs and various biologically active substances were well preserved in the fish scale scaffolds. In addition, the surface of fish scales retained the oriented collagen nanofibers after decalcification, which provided a suitable environment for the oriented growth of cells. Furthermore, the anisotropic fish scale scaffold containing large amounts of collagen nanofibers also contributes to cell adherence and proliferation of HUVECs and MSCs. These characteristics gave fish scales the potential to become an excellent 3D tissue engineering scaffold by combining the exclusive advantages of synthetic and natural materials, respectively. In in vivo animal study, MSCs‐loading fish scale scaffolds were observed to greatly reduce the area of necrosis caused by ischemia of the distal flap, effectively suppressed the inflammatory response and promoted the regeneration of capillaries around the implantation site. Based on these striking features, we expect these cell loading fish scales, as anisotropic structural biomaterials with high compatibility and degradability, will have more application value in cell delivery and tissue regeneration.

## 4 Experimental Section

### Materials

Tilapia scales obtained from Jinxianghe Road Market (Najing). Sodium lauryl sulfate, nitric acid, Live Cell Staining Kit Calcein‐AM/PI were purchased from Shanghai Yanhui Biological Technology Co., Ltd.. Decalcification Endpoint Detection Kit was provided by Beijing Ita Biology. The Cells from the standard fibroblast line (NIH 3T3) and HUVECs were obtained from Beijing BioRich. 2 months SD rats were purchased from the Animal Management Department of Shanghai Family Planning Research Institute (SPF). All animal experiment procedures were carried out after passing the experimental animal ethics review of the Affiliated Drum Tower Hospital of Nanjing University (approval number 2021YFA1015300).

### Decellularization of Fish Scales

Fresh fish scales from Tilapias were harvested and cleaned by phosphate buffered solution (PBS). The washed fish scales were repeatedly frozen and thawed at −80 ℃ for four times, and then soaked in 0.1% NaOH solution and 3% H_2_O_2_ solution in order to remove residual fish skin, fat, and some impurities. Then, the fish scales were further immersed in 2% SDS to remove cells. Finally, the fish scales were sterilized by ethylene oxide and stored in PBS solution.

### Decalcification of Fish Scales

The decalcification process was carried out by nitric acid. Briefly, the decellularized fish scale was immersed in 5% nitric acid. The degree of decalcification was determined by the decalcification endpoint kit. Then the fish scales were further decalcified by 10% EDTA for 2 days to totally remove the Calcium.

### Histology and Immunostaining

Fish scale samples were dehydrated in gradient ethanol (55–100%), and then fixed in formalin and embedded into paraffin block. Paraffin sections (7 µm) were stained with hematoxylin and eosin to determine the residual cells. Masson staining and Sirius red staining were used to determine the collagen structure, and Alcian blue staining was used to detect GAG content.

### Mechanical Performance Test

Mechanical test was performed in an electronic tensile machine (Shenzhen Sansi aspect Technology Co., Ltd.). Fish scales were made into dog‐stick shape tensile specimens (15×3 mm) in longitudinal and transverse orientation, respectively. The stretching length was about 10 mm, and the stretching speed was 5 mm s^−1^.

### Biocompatibility of Prepared Fish Scale

In order to explore the effect of nitrate decalcification on fish scale, materials with different decalcification durations were inoculated with 3T3 cells and cultured for 1 day. Briefly, ≈4000 3T3 were seeded on the scaffold and cultured in a 96‐well plate. After removal of the medium, 10% MTT solution was added and cultured for 4 h. Then, Dimethyl sulfoxide was used for dissolving the crystals. 100 µL liquid from each well was prepared for the measure of the OD value on a plate reader. OD value of the control group was set as 100% to for quantitative analysis of cell viability.

### Preparation of MSCs‐Loading Fish Scale

The scaffold was soaked in ethanol for half an hour, and then washed with PBS buffer. The washed scaffolds and well plates (substrates used as negative controls) were UV sterilized for 1 day, and immersed in normal cell culture medium for 1 day. Then the medium was removed and MSCs cells were seeded (1×10^4^ cells per sample), and cultured in a humidified atmosphere of 5% carbon dioxide for 1, 2, and 3 days.

### In Vitro Viability and Proliferation Ability Test

In order to detect the survival of endothelial cells and MSCs on fish scale. Fish scale was prepared and seeded with 10^4^ endothelial cells and MSCs, respectively. The Calcein staining showed the number of viable cells. The aforementioned MTT method was used to detect the viability of cells at 1, 2, and 3 days. For cell morphology and attachment of MSCs, phalloidin was used to stain actin filaments. Briefly, the fish scale cultured with MSCs for three days, then soaked in paraformaldehyde (4%) for half an hour and washed with PBS, blocked with 5% BSA for half an hour and added with phalloidin staining solution (1:400). In order to count the cell orientation, 25 cells stained by Phalloidin were randomly selected for the direction statistics.

### Degradability Test of Fish Scales

To detect the degradability, the prepared fish scale and PEGDA hydrogel film samples were implanted under the skin of the SD rat's back. The rats were anesthetized after 1 month and the skin of the marked part was taken out to observe whether there was any remaining material left.

### In Vivo Animal Experiments

16 SD male rats (around 200 g) were randomly divided into four groups: control group, FS group, MSCs group, and MSCs‐loading fish scale group. The rats were anesthetized by using a small animal respiratory anesthesia machine, and then shaved with electric scissors to remove the hair on the back. Then a random flap of about 5×1.5 cm was opened on the back of the rat. The control group was washed with PBS only. The FS group only implanted with Fish scale scaffold, the MSCs groups was injected about 10^6^ cells. To fabricate MSCs‐loading FS scaffold, about 5 × 10^5^ cells were dripped on the FS scaffold, after 2 h the same number of cells were seeded on the other side of the scaffold. 7 days after operation, the area of the skin flap necrosis was measured and used a thermal imaging analyzer to further determine the size of the necrosis skin flap.

### In Vivo Survival Time

In order to evaluate the survival rate of MSCs in vivo, mice were divided into two groups: MSCs injection group and MSCs‐loading fish scale scaffold group. MSCs with green fluorescence were used for experiments, and IVIS (R) imaging system was used for signal analysis at 2 h and 7 days after surgery.

### Immunohistochemistry Test

The rats were euthanized by inhaled an overdose of anesthesia, and skin flap samples were collected. Collect skin flaps and the surrounded tissues, fix them in 3% glutaraldehyde. The tissue morphology and structure of each rat flap was assessed by H&E staining. CD31 immunohistochemistry was used to detect the number of microvessels, CD68 was used to detect macrophage density, iNOS+ was used to detect the number of M1 type macrophages and CD206 staining was used to detect the number of M2 type macrophages.

### Characterization

The fish scales before and after the treatment were observed by light microscope. And the images were taken with a stereo microscope (JSZ6S, Shanghai Dianying Optical Co., Ltd., Shanghai, China), and equipped with a CCD camera (Optical Camera, China). Scanning electron microscope (SEM) observed the micro–nano structure changes of fish scale before and after the treatment. The water contact angle was measured by SDC‐100 contact angle measuring instrument. The Live/Dead Cell Double Staining Kit (Guangzhou Weber Technology Co., Ltd.) and Alexa Fluor 555 labeled phalloidin (Shanghai Liji Biological Technology Co., Ltd.) was used to observe the growth of cells on the material. The smart phone was used to take pictures to observe the residual condition of the implanted material, and the FTIR thermal imaging device recorded Skin flap necrosis.

### Statistical Analysis

The processed data was expressed as mean ± SD. SPSSAU 24.0 was used to evaluate statistical significance. An independent sample *t* test was used to assess the significant difference between the control and experimental groups. Choose *p* <0.05 as the significance level, * means 0.01< *p* <0.05. ** means *p* <0.01.

## Conflict of Interest

The authors declare no conflict of interest.

## Author Contributions

Y.J.Z. conceived the idea and designed the experiment; X.L. conducted experiments and data analysis; B.K. and Y.J.Z. assisted with cell culture; X.L. and Y.J.Z. wrote the manuscript. Y.J.Z. supervised the manuscript.

## Supporting information

Supporting Information

## Data Availability

The data that supports the findings of this study are available in the supplementary material of this article.
