# Supplementary material for: Bioactive Fish Scale Scaffolds with MSCs‐Loading for Skin Flap Regeneration
Source: Adv Sci (Weinh). 2022 May 22;9(21):2201226. doi: 10.1002/advs.202201226 (PMC9313489; doi:10.1002/advs.202201226)
Supplement: Supplementary file 1 — Supporting Information [file ADVS-9-2201226-s001.pdf]

ADVANCED SCIENCE
Open Access

Supporting Information

for *Adv. Sci.*, DOI 10.1002/advs.202201226

Bioactive Fish Scale Scaffolds with MSCs-Loading for Skin Flap Regeneration

*Xiang Lin, Bin Kong, Yujuan Zhu and Yuanjin Zhao**

Supplementary

Bioactive fish scale scaffolds with MSCs-loading for skin flap regeneration

Xiang Lin [1], Bin Kong [1], Yujuan Zhu [2], Yuanjin Zhao [1,2,3,*]

[1] Department of Rheumatology and Immunology, Nanjing Drum Tower Hospital, School of Biological Science and Medical Engineering, Southeast University, Nanjing 210096, China

[2] Oujiang Laboratory (Zhejiang Lab for Regenerative Medicine, Vision and Brain Health), Wenzhou Institute, University of Chinese Academy of Sciences, Wenzhou 325001, China

[3] Institute for Stem Cell and Regeneration, Chinese Academy of Science, Beijing 100101, China

Email: yjzhao@seu.edu.cn

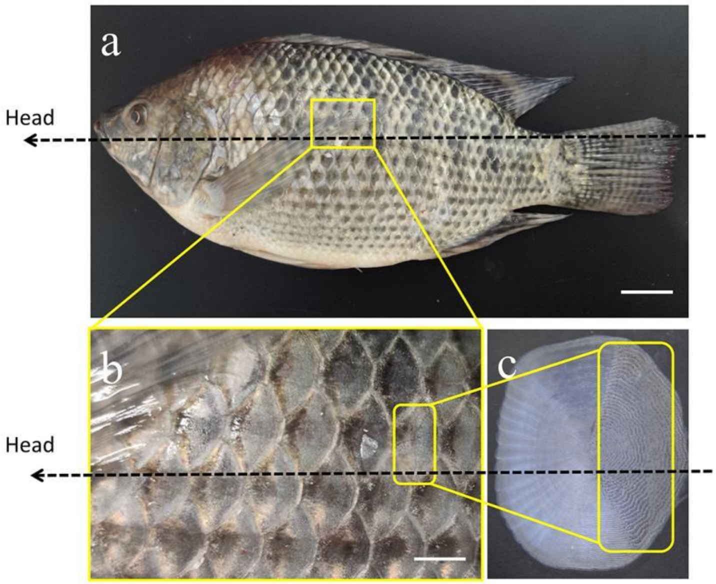

Figure S1. (a) Image of tilapia scales, the scale bar is 2 cm; (b) Magnified image of fish scale, the scale bar is 2cm; (c) Embedded part of fish scale under the dermis.

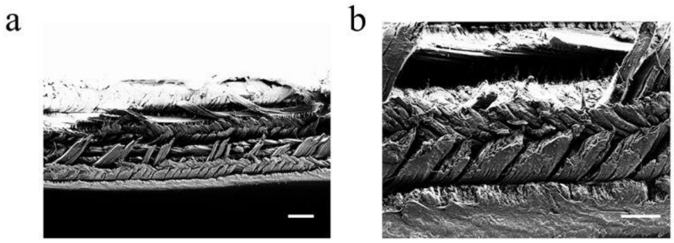

Figure S2. (a) Cross-sectional SEM image of fish scale, the scale bar is 20 μm; (b) The magnified image of cross-sectional part, the scale bar is 5 μm.

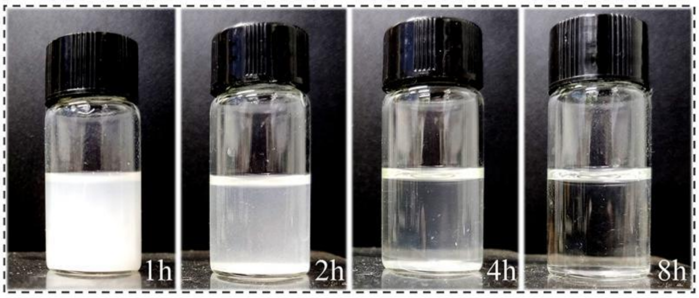

Figure S3. Images of calcium oxalate precipitation after decalcification at different time points.

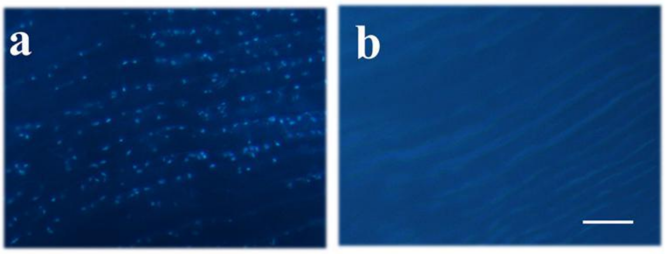

Figure S4. (a) The DAPI staining of the fish scale before (a) and after (b) decellularization, the scale bar is 100 μm.

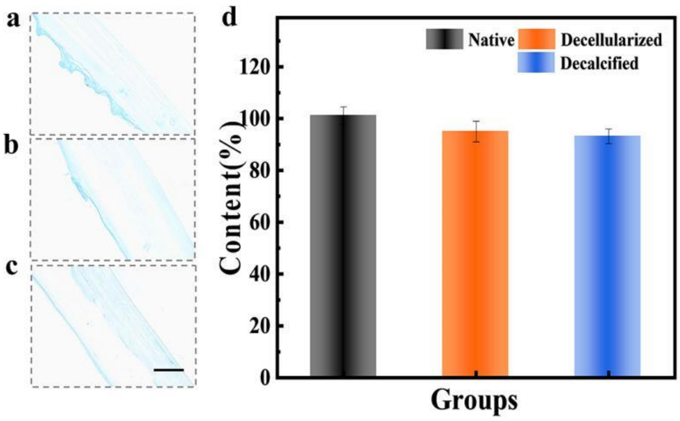

Figure S5. Alcian blue staining images of (a) native group, (b) decellularized group, (c) decalcified group, the scale bar is 200 μm; (d) Statistical analysis of GAGs content in different groups, the native group was set as 100 % (n=4). The error bar represents standard deviation.

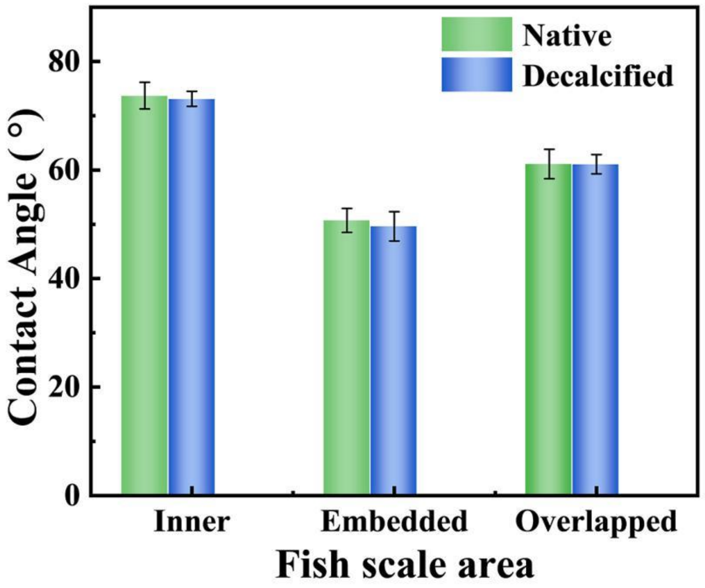

Figure S6. Water contact angle of different parts before and after decalcification.

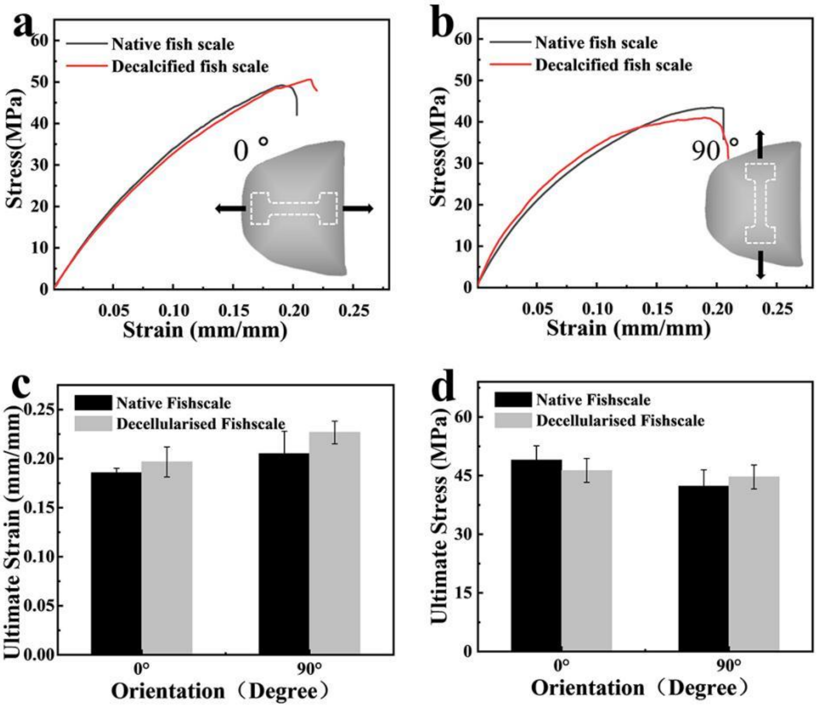

Figure S7. Stress-strain curves of different fish scale samples in (a) 0 ° and (b) 90 °; The ultimate strain of fish scale samples in different orientation (c); (d) The ultimate stress of fish scale samples in different orientation (n=5). The error bar represents standard deviation.

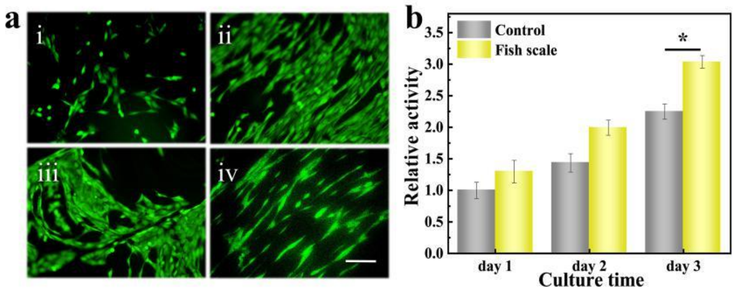

Figure S8. (a) Fluorescence images of HUVECs cultured on (i) petri dish, (ii) and (iii) overlapped area, (iv) inner surface of the fish scale, the scale bar is 100 μm; (b) Cell

proliferation activity of HUVECs on different materials (n=5). The error bar represents standard deviation.

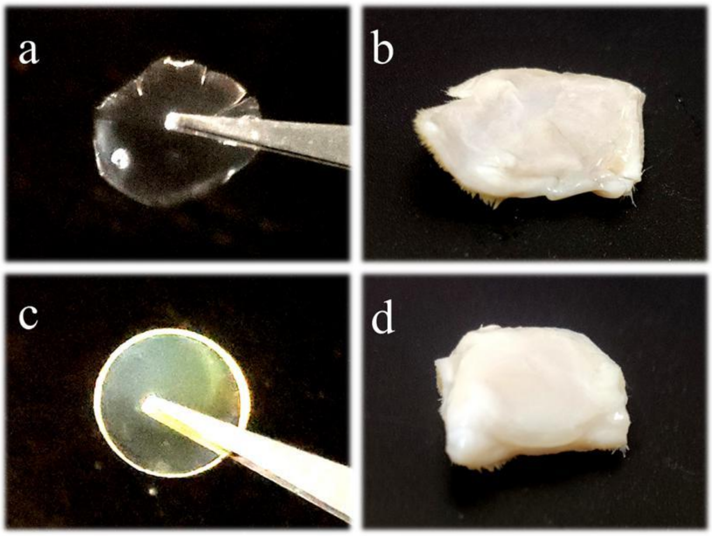

Figure S9. Optimal image of fish scale (a) after decalcification and (b) implanted for one month; Optimal image of PEGDA hydrogel (c) before implantation and (d) implanted for one month.

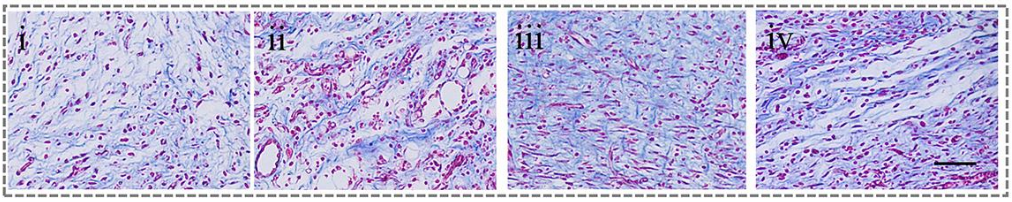

Figure S10. Representative masson staining images of i) control groups, ii) FS group, iii) MSCs group, iv) MSCs&FS group, the scale bar is 100 μm.

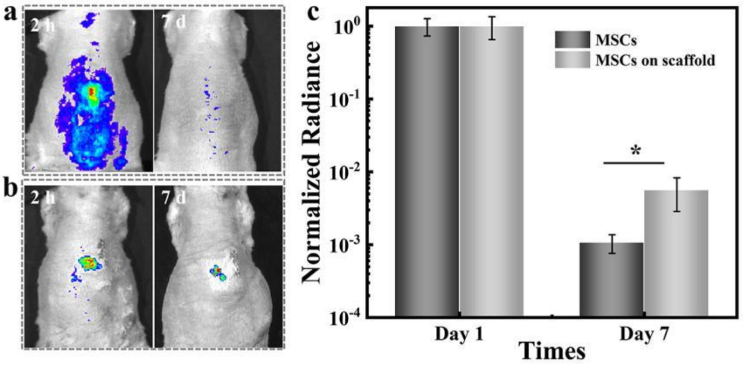

Figure S11. (a) Fluorescence signals after subcutaneous injection of MSCs; (b) Fluorescence signals after the implantation of MSCs-loading fish scale scaffolds; (c) Quantitatively study of the normalized radiance signals in different groups (n=4). The error bar represents standard deviation. * $p < 0.05$.

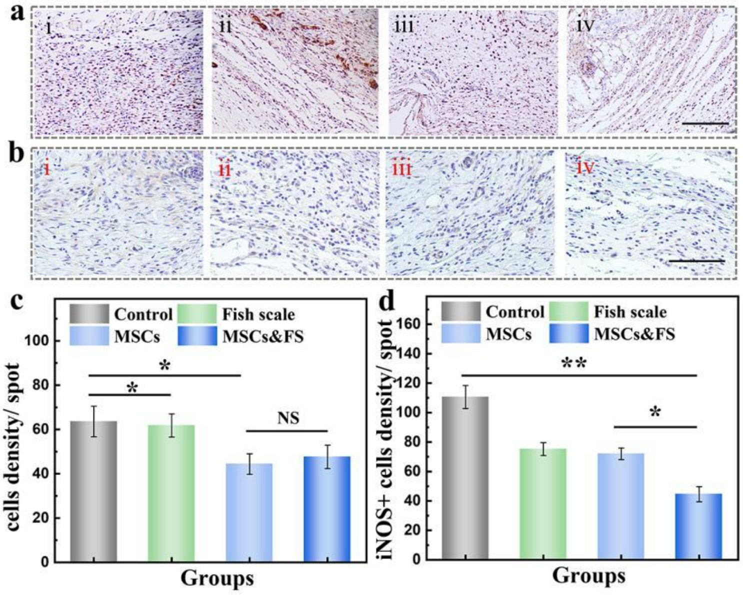

Figure S12. (a) Representative CD68 staining images of i) control groups, ii) FS group, iii) MSCs group, iv) MSCs&FS group, the scale bar is 100 μm; (b)

Representative iNOS+ staining images of i) control groups, ii) FS group, iii) MSCs group, iv) MSCs&FS group, the scale bar is 100 μm; (c) The corresponding cell density per spot. (d) The corresponding iNOS+ cell density per spot. The error bar represents standard deviation, NS: no significant, * $p < 0.05$. ** $p < 0.01$.
